# *Ureaplasma parvum* and *Ureaplasma urealyticum* induce distinct types of inflammation in neonates and human epithelial cell models

**DOI:** 10.1038/s41390-025-04415-0

**Published:** 2025-10-03

**Authors:** Hongzhen Zhu, Pol Oliveras-Julià, Gea F. Hasperhoven, Luca L. van Leeuwen, Ad C. J. M. de Bruijn, Marijn C. Verwijs, Annemarie M. C. van Rossum, René F. Kornelisse, Kim Stol, Wendy W. J. Unger

**Affiliations:** 1 Department of Pediatrics, Laboratory of Pediatrics, Erasmus MC University Medical Center - Sophia Children’s Hospital, Rotterdam, The Netherlands; 2 Department of Neonatal and Pediatric Intensive Care, Division of Neonatology, Erasmus MC University Medical Center - Sophia Children’s Hospital, Rotterdam, The Netherlands; 3 Department of Pediatrics, Division of Pediatric Infectious Diseases and Immunology, Erasmus MC University Medical Center - Sophia Children’s Hospital, Rotterdam, The Netherlands; 4 Department of Pediatrics, Division of Pediatric Infectious Diseases and Immunology, Radboud University Medical Center, Amalia Children’s Hospital, Nijmegen, The Netherlands

## Abstract

**Background:**

Development of bronchopulmonary dysplasia (BPD) in premature neonates is associated with infection and inflammation. Both *Ureaplasma parvum* and *Ureaplasma urealyticum* are associated with BPD. We examined whether there is a difference in pathogenicity between the two species

**Methods:**

Tracheal aspirates of 25 preterm neonates were analyzed for bacterial presence and inflammatory mediators. Alveolar epithelial cells were infected with *U. parvum and U. urealyticum* strains to assess inflammatory mediators, cell death and oxidative stress.

**Results:**

*U. parvum* was detected in 2/25 and *U. urealyticum* in another 3/25 neonates. *E. coli* was co-detected in 3/5 *Ureaplasma*-positive samples. *U. parvum*-positive samples contained high IL-6, IL-8 and CXCL5. *U. urealyticum*-positive samples also contained high IL-6 and IL-8, but low CXCL5, and high CXCL1 and CCL2. Five-to-ten-fold higher IL-6 and two-fold higher IL-8 levels were detected in *U. parvum*-infected cell cultures than *U. urealyticum*, whereas apoptotic cell death was detected in *U. urealyticum-*infected cultures. Infection with both species induced ROS.

**Conclusion:**

Both *Ureaplasma* species may contribute to inflammation and cell damage, via oxidative stress, as observed in BPD, yet through different mechanisms. *U. parvum* infection induces a strong pro-inflammatory mediator response in alveolar epithelial cells while *U. urealyticum* infection results in cell death.

**Impact:**

- *U. urealyticum* and *U. parvum* can contribute to the inflammation and cell damage seen in chronic lung disease through the secretion of inflammatory mediators.
- The two species differ in their mechanism of action: *U. parvum* infection induces a strong pro-inflammatory mediator response in alveolar epithelial cells while *U. urealyticum* infection results in epithelial cell death.
- Our data provide new insights into the role of *Ureaplasma* in the development of chronic lung disease in premature infants.

## Introduction

*Ureaplasma* spp. are atypical bacteria that belong to the *Mycoplasmatacae* family, which members are marked by the lack of a cell wall.^1^ The absence of a cell wall has implications for treatment, as it makes *Ureaplasma* spp. insensitive to beta-lactam antibiotics. Human *Ureaplasma* has been separated into two species with 14 known serovars: *U. parvum* includes serovars 1, 3, 6, and 14, while *U. urealyticum* includes serovars 2, 4, 5, 7–13.^2–4^

*Ureaplasma* spp. commonly colonize the mucous membranes of the lower urogenital tracts in both asymptomatic men and women, with about 40% of genitourinary samples from these individuals testing positive.^1,5^ In pregnant women, *Ureaplasma* spp. can ascend and cause chorioamnionitis, preterm labor, and preterm birth.^6–10^ Both animal and clinical studies have shown that intra-amniotic *Ureaplasma* spp. infections can contribute to preterm labor and delivery. For example, intra-amniotic inoculation of *U. parvum* clinical isolates in pregnant mice and sheep resulted in preterm births.^11–13^ Similarly, in humans, the intra-amniotic presence of *U. urealyticum* strongly correlated with preterm labor and preterm delivery.^14,15^ Vertical transmission of *U. urealyticum* from mother to fetus can also occur in utero, with the fetus being exposed to the bacteria through aspiration of amniotic fluid, thereby directly exposing fetal lungs to *Ureaplasma* spp.^11,16,17^ This has been observed in animal studies, where *Ureaplasma* spp. were isolated from the lungs of newborn mice and lambs.^11–13^ *Ureaplasma* spp. have also been isolated from respiratory tract samples of human preterm newborns, such as tracheal aspirates and bronchoalveolar lavage samples.^2,11,18,19^ The rates of prenatal and perinatal transmission, as well as subsequent fetal colonization and infection, appear to be inversely proportional to gestational age.^20^

*Ureaplasma* spp. in neonatal lungs is linked to inflammation and disruptions in lung development, potentially leading to bronchopulmonary dysplasia (BPD), a common complication in premature infants and a major cause of life-long morbidity.^21–25^ BPD is characterized by diffuse structural lung damage, neutrophilic inflammation, and fibrosis of the parenchyma of the neonatal lungs.^26^ The pathogenesis of BPD suggests that lung injury is frequently initiated by intra-uterine infection and a dysregulated inflammatory response.^11,16,27^

It is hypothesized that the presence of *Ureaplasma* spp. in the neonatal lungs can result in an augmented inflammatory response that contributes to the development of BPD. Lambs born prematurely upon intra-uterine *Ureaplasma* spp. injection, showed *Ureaplasma* infection in the developing lung, with increased levels of IL-1β, IL-6, and IL-8, as well as neutrophil and monocyte infiltration.^28–30^ Similar neutrophil and monocyte/macrophage influx and increased levels of inflammatory TNFα were observed in the lungs of *U. urealyticum-*positive preterm neonates.^31,32^ Additionally, in vitro and in vivo studies demonstrated that *Ureaplasma* spp. induces apoptosis in human type II lung epithelial cells, human pulmonary microvascular endothelial cells, and macrophages, contributing to cellular damage and pulmonary fibrosis.^24,33–35^ Studies in preterm infants and animal models show evidence of interstitial fibrosis and cell apoptosis in *Ureaplasma* infected lungs, supporting the idea that *Ureaplasma* infection worsens the inflammatory environment, potentially exacerbating BPD.^24,30–33^

Although studies indicated an association between the presence of *Ureaplasma* spp. and BPD development^36^ a species-specific contribution remains unclear due to differences in study populations and methods. Some studies highlight *U. parvum*,^18,22^, others *U. urealyticum*,^14,15,19^, while others find no difference.^37,38^ In addition, limited in vitro data exist on qualitative and quantitative differences in host cell responses between *Ureaplasma* species. To address this, we compared the pulmonary pathogenicity and host responses of the two *Ureaplasma* species, *U. urealyticum* and *U. parvum*, using neonatal tracheal aspirates and a human lung epithelial cell infection model.

## Materials and methods

### Clinical background

Tracheal aspirate samples were collected randomly from intubated patients at the Neonatal Intensive Care Unit (NICU) of a tertiary care facility between April 2019 and May 2024. To optimally ventilate intubated neonates, removal of obstructive sputum by rinsing the tube and trachea with 1–2 mL NaCl 0.9% with subsequent aspiration of fluid is part of standard care. These tracheal aspirates were collected in a sterile tube and transported to the laboratory. Aspirates were processed immediately, and supernatants were stored in a freezer until further analysis. The study was approved by the hospital’s Medical Research Ethics Committee (ID MEC-2020-0159). Parents provided informed consent to use leftover materials, including tracheal aspirates.

### Detection of pathogens in tracheal aspirates

Total DNA was extracted from 200 μL tracheal aspirates, that were spiked with a known concentration of seal herpesvirus 1 (PhHV-1) DNA to control for nucleic acid extraction efficiency. Presence of bacteria was assessed using a single-plex PCR with pan-bacterial 16S primers. *Ureaplasma* species, *E. coli*, *K. pneumoniae*, and *S. agalactiae* (Group B Streptococcus) were identified by multiplex quantitative real-time PCR using the following reaction mixture: 10 μL iQ multiplex powermix (Bio-Rad), 20x primer/probe mix, and 5 μL DNA template in a total volume of 20 μL. *S. aureus* was detected by quantitative real-time PCR single-plex: 10 μL Taqman universal master mix, 0.5 µM primers, 0.25 µM probe, and 1 µL DNA template were mixed in a total volume of 20 µL. The PCR cycling conditions were 2 min at 95 °C; 40 cycles of 15 s at 95 °C, 1 min at 60 °C. The primer and probe sequences are shown in Table S1.

### *U. urealyticum* and *U. parvum*

*U. urealyticum* and *U. parvum* reference strains were obtained from the American Tissue Culture Collection (ATCC); the strains are as follows: *U. urealyticum* #1-ATCC 27618 (formerly 50782 T or 50912, serovar 8), *U. urealyticum* #2-ATCC 27817 (serovar 5), *U. parvum* #3-ATCC 27815 (serovar 3), *U. parvum* #2-ATCC 700970 (serovar 3). *U. urealyticum* #3-2983, *U. parvum* #1-2990, and *U. parvum* #4-3763 are clinical isolates from pregnant women (kind gift of Dr. Biernat Sudolska; Jagiellonian University Medical College, Krakow, Poland). *Ureaplasma* isolates were cultured in modified 10B broth (referred to as “m10B broth”) containing 2% PPLO (pleuropneumonia-like organism medium; Becton, Dickinson & Company, Franklin Lakes, NJ), 2.2% Yeast extract (Becton, Dickinson & Company, Franklin Lakes, NJ), 20% (v/v) heat-inactivated horse serum (Bio-West; Nuaillé, France), 0.4% L-Cysteine-HCl (Sigma; Steinheim, Germany), 4% urea (VWR; Solon), 1.6% Putrescine dihydrochloride (Sigma), 0.025% phenol red, 0.5% Isovitalex Enrichments (BD) and 10^5^ U/100 mL Penicillin (Hospital Pharmacy, Erasmus MC) were dissolved in MilliQ; the medium was adjusted to pH 6.0. After filtration through a 0.22 μm membrane (Millipore 0.22 µM Vacuum filter system), the medium was adjusted to pH 6.5. *Ureaplasma* strains were cultivated in m10B broth at 37 °C with 5% CO_2_ until the medium changed from yellow to pink-red. The *Ureaplasma* were harvested, and aliquots were stored at −80 °C until use. The bacterial concentrations of the suspensions were confirmed with colony-forming units (CFU) onto mA8 agar plates. To generate mA8 agar plates, first mA8 basal medium was prepared: 7.5 g PPLO, 2.5 g tryptic soy broth, 2 g NaCl, 1 g KH₂PO₄, 7 g agar, and 385 mL Milli-Q water were mixed (pH 6.0). After sterilization, the basal broth was cooled to 55 °C, and the following supplements were added: 5 ml yeastolate (0.25 g/mL); 100 mL heat-inactivated horse serum; 0.5 mL filter-sterilized L-cysteine (0.1 g/mL); 1.25 mL filter-sterilized urea (0.4 g/mL); 2.5 mL isovitalex; 5 mL filter-sterilized putrescine (0.2 g/mL); 2.5 mL Penicillin (2×10^5 ^U/mL) and 0.5 mL filter-sterilized CaCl₂·2H₂O (0.16 g/mL).

### Heat inactivation of *Ureaplasma* species

An aliquot of *Ureaplasma* stock was resuspended in PBS/2 mM MgCL_2_, diluted to 1 × 10^7^ CFU/mL, and incubated at 56 °C for 45 min. After cells were cooled to RT, the *Ureaplasma* bacteria were centrifuged at 13,000 × *g* for 10 min. The supernatants were removed, and the pellets were washed twice with PBS, then resuspended in broth and homogenized. Heat-inactivated *Ureaplasma* bacteria were subsequently stored at –80 °C until further use.

### *Ureaplasma* infection of human alveolar epithelial cells

Human alveolar epithelial cells (ATCC, CCL-185) were cultured at 37 °C and 5% CO_2_ in RPMI-1640 medium (Thermo Fisher Scientific) supplemented with 10% heat-inactivated fetal bovine serum (RPMI/10% FCS) in the absence of antibiotics. The cells were seeded in a 96-well flat-bottom plate (50,000 cells/well; Greiner, Frickenhausen, Germany) and grown to 80% confluence at 37 °C and 5% CO_2_. The cells’ confluent monolayer was confirmed by observation with a Nikon TMS inverted microscope (Hicksville, NY). Aliquots of *Ureaplasma* bacteria were diluted in RPMI-1640 medium to a concentration of 5 × 10^5^ and 5 × 10^6^ CFU/mL to reach multiplicities infection (MOI) 1 and 10, respectively. The epithelial cells were initially washed with PBS and then infected with live *U. urealyticum* or *U. parvum*, as well as heat-inactivated *U. urealyticum* or *U. parvum*, at different MOI for 24 h at 37 °C and 5% CO_2_. Cells were incubated with medium or FSL-1 as negative and positive controls, respectively. All experimental conditions were performed in triplicate.

### *Ureaplasma* infection of alveolar epithelial cells in the presence of *E. coli* LPS

Alveolar epithelial cells were cultured with indicated *U. urealyticum* or *U. parvum* strains at MOI 10 or MOI 100 in the absence or presence of indicated concentrations of LPS (LPS from *Escherichia coli* O5:B55, InvivoGen, France). After 24 h of culture, supernatants were harvested, and the levels of IL-6 and IL-8 were determined by ELISA. Additionally, after 48 h of culture, the cell damage was determined by quantifying the amounts of lactate dehydrogenase (LDH) released into the supernatants.

### Determination of cytokine concentrations by ELISA and LEGEND-plex assay

Interleukin (IL)-6, IL-8, IL-1β, IL-10 and tumor necrosis factor (TNF)-α concentrations in the supernatants were measured by ELISA using specific antibody pairs (Thermo Fisher Scientific, Waltham, MA; BD Biosciences, Heidelberg, Germany), according to the manufacturer’s instructions. 3, 3′, 5, 5′-Tetramethybenzidine (Sigma-Aldrich) was used as a substrate. The optical density was measured at OD_450nm_ using a microplate reader (SpectraMax iD3, Molecular Devices, San José, CA). A Human Proinflammatory Chemokine Panel 1 (BioLegend, San Diego, CA) was used to measure the concentration of MCP-1 (CCL2), RANTES (CCL5), IP-10 (CXCL10), Eotaxin (CCL11), TARC (CCL17), MIP-1α (CCL3), MIP-1β (CCL4), MIG (CXCL9), MIP-3α (CCL20), ENA-78 (CXCL5), GROα (CXCL1), I-TAC (CXCL11) and IL-8 (CXCL8) in the supernatants, according to the manufacturer’s instructions. The samples were acquired using a FACSCanto™ II flow cytometer (BD Bioscience, Heidelberg, Germany). Data were analyzed using LEGENDplex V8.0 Data Analysis Software (Biolegend, San Diego, CA).

### Evaluation of cell death

Human alveolar epithelial cells were seeded in a 96-well flat-bottom plate at a density of 50,000 cells per well or on 8-well cell culture slides (MatTek, Ashland, MA) and grown to 80% confluence at 37 °C and 5% CO_2_. The confluent epithelial monolayers were gently washed with PBS, and live or heat-inactivated *U. urealyticum* or *U. parvum* were added to the cells at MOI 100. A549 cells incubated with medium or 10% DMSO were used as negative and positive control, respectively. After 24 h or 48 h, supernatants were harvested to determine the amounts of LDH released by damaged cells using the CyQUANT LDH Cytotoxicity Assay kit (Thermo Fisher Scientific). The percentage of damaged epithelial cells was calculated according to the manufacturer’s instructions. To evaluate induction of apoptosis, cells were stained with Annexin-V antibody (Thermo Fisher Scientific), imaged, and quantified with ImageJ. Additionally, cells were washed with PBS and incubated with the LIVE/DEAD fixable Violet Dead Cell Stain (Thermo Fisher Scientific) for 30 min at 4 °C, and the frequency of dead cells was assessed using Canto-II flow cytometry (BD Bioscience, Heidelberg, Germany). A minimum of 50,000 events was acquired and analyzed with FACSDiva 8.0.1 software (BD Bioscience).

### Evaluation of oxidative stress markers–Reactive Oxygen Species (ROS), Glutathione Peroxidase (GSH-Px) activity, and Malondialdehyde (MDA) concentration

To assess ROS activity, A549 cells were seeded at 50,000 cells/well onto black, clear-bottom 96-well plates in phenol red free media and incubated for 24 h at 37 °C with 5% CO_2_. Cells were stained with DCFDA for 45 min. After washing with PBS, live or heat-inactivated *U. urealyticum* or *U. parvum* were added to the cells at MOI 10 for 3 h. As a positive control of ROS induction, cells were exposed to 200 µM hydrogen peroxide. ROS activity was detected by fluorescence spectroscopy with excitation/emission at 485/535 nm and expressed as fluorescence intensity.

GSH-Px activity and MDA concentration were assessed using commercial kits (Thermo Fisher Scientific). In brief, A549 cells were seeded at 50,000 cells/well in 96 well flat-bottom plates and incubated for 24 h at 37 °C with 5% CO_2_. *U. urealyticum* or *U. parvum* were added to the cells at MOI 100. After 24 h incubation, the cells were washed with PBS, incubated with lysis buffer, and GSH-Px and MDA were assessed according to manufacturer’s instructions. GSH-Px activity is expressed as U/mg protein.

### Statistical analysis

Statistical analyses were performed using Prism 9.0 (GraphPad Software Inc., San Diego, CA). Data are presented as means ± standard error of the mean (SEM) or standard deviation (SD) from one experiment, as indicated with usually three technical repeats within each experiment. Experiments were repeated at least twice. Continuous variables are presented as mean and standard deviation, or median and interquartile range (IQR). Data were checked for normality, and statistically significant differences between groups were determined by one-way ANOVA with post-hoc Dunnett’s multiple comparisons test. Results were considered statistically significant when *p* < 0.05.

## Results

### Incidence of *Ureaplasma* in tracheal aspirates of preterm neonates

A total of 25 tracheal aspirate samples from 25 preterm infants (gestational age <36 weeks) admitted to the NICU were collected for this exploratory study. Demographic data are summarized in Table 1. The median gestational age of these patients was 26 weeks+ 5 days (interquartile range (IQR) 25 weeks + 4 days – 29 weeks + 1 days) and birth weight was 910 g (IQR 670–1290 g). The median number of days on a ventilator was 10 days (IQR 5–21 days). Of the 25 neonates, 18 developed BPD. Bacteria were detected in all samples, with targeted analysis revealing the presence of *Ureaplasma* spp., *Klebsiella pneumoniae*, *Staphylococcus aureus*, and *Escherichia coli*. In none of the samples GBS was detected. *Ureaplasma* spp. were detected in five samples: two were positive for *U. parvum*, and three were positive for *U. urealyticum* (Table 1). *K. pneumoniae* and *S. aureus* were detected in six (33.3%) and eight (44.4%) samples respectively. More than one pathogen was detected in 13 (52%) samples (Fig S1). In *Ureaplasma*-positive infants, the median gestational age was 26 weeks+ 5 days (IQR 26 weeks + 2 days − 30 weeks + 3 days) and birth weight was 950 g (IQR 577.5−1655 g). Only the number of days on ventilation was higher in neonates who were *Ureaplasma*-positive compared to all neonates (19 days; IQR 8.5–67.75 days versus 10 days; IQR 5–21 days). Notably, four out of five *Ureaplasma*-positive infants developed BPD. Of the investigated pathogens, only *E. coli* (60%, 3/5 cases) was detected in *Ureaplasma*-positive patients.

**Table 1:** Characteristics of the neonates enrolled in the present study

Neonates’ characteristics	All	*Ureaplasma*-positive	*Ureaplasma*-negative
Tracheal aspirate (*n*)	25	5	20
Gestational age (median; IQR) (weeks + days)	26 + 5 (25 + 4 – 29 + 1)	26 + 5 (26 + 2 – 30 + 3)	27 + 2 (25 + 3 – 29 + 1)
Birth weight (median; IQR) (gram)	910 (670–1290)	950 (577.5–1655)	905 (717.5–1310)
Male (*n*, %)	10 (40%)	4 (80%)	6 (30%)
Female (*n*, %)	15 (60%)	1 (20%)	14 (70%)
BPD (*n*, %)	18 (72%)	4/5 (80%)	14/20 (70%)
Days of ventilation (median; IQR) (days)	10 (5–21)	19 (8.5–67.75)	9.5 (4.75–22.5)
Tracheal aspirate bacteria (*n*, %)			
Any bacteria	25 (100%)	5 (100%)	20 (100%)
*U. urealyticum*	3 (12%)	3 (60%)	0
*U. parvum*	2 (8%)	2 (40%)	0
*GBS*	0	0	0
*E. coli*	19 (76%)	3 (60%)	16 (80%)
*K. pneumoniae*	6 (24%)	0	6 (30%)
*S. aureus*	8 (32%)	0	8 (40%)

*BPD* bronchopulmonary dysplasia, *IQR* interquartile range.

### Inflammatory mediators in *Ureaplasma*-positive tracheal aspirates

**Fig. 1:**
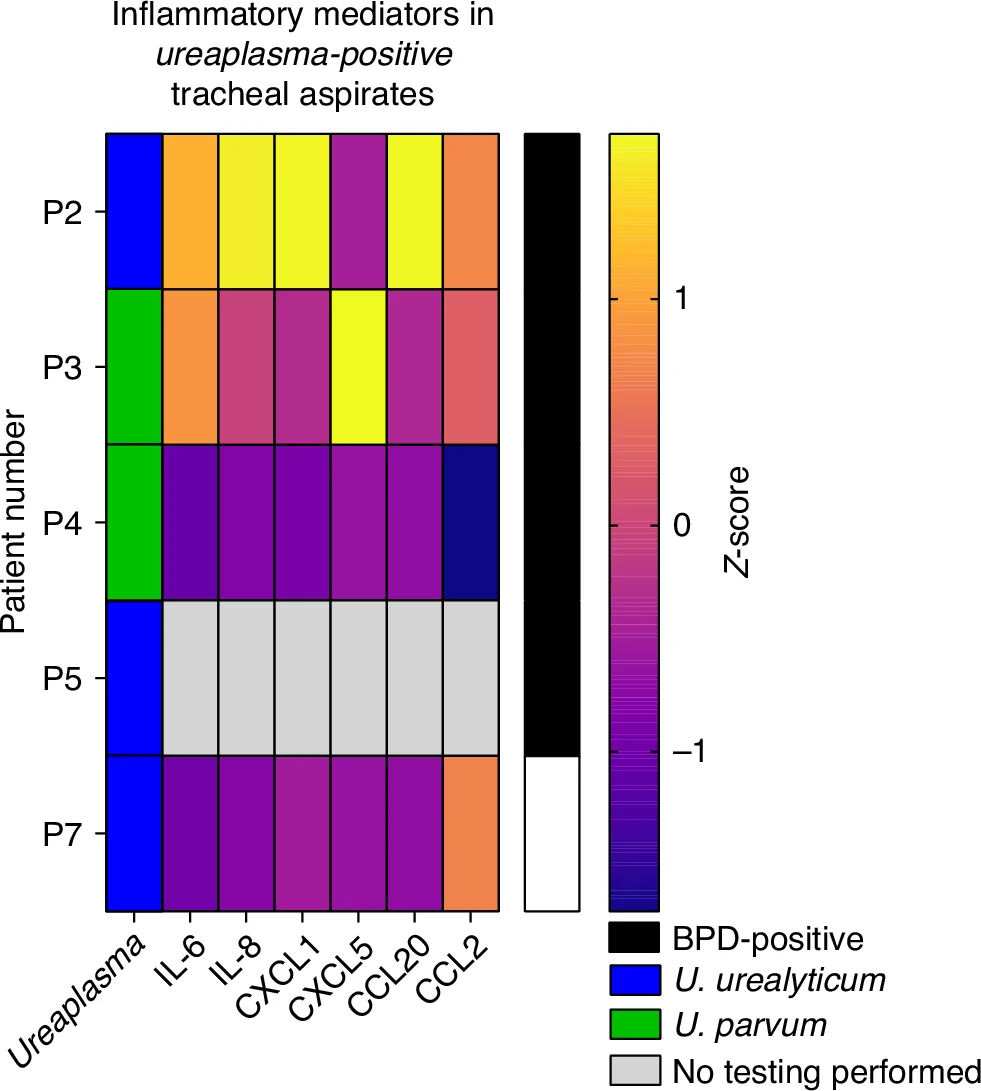
Comparison of inflammatory mediators in *Ureaplasma*-positive tracheal aspirates. Heatmap showing changes in abundances of six quantified cytokines and chemokines in tracheal aspirates from five *Ureaplasma*-positive preterm neonates. Pro-inflammatory cytokines and chemokines were determined by ELISA and Legend-plex assay. Individual protein abundance per milliliter of tissue was *z*-score normalized. Blue indicates lower protein concentrations while yellow represents higher protein concentrations (see scale). Dark blue squares denote the presence of *U. urealyticum* in a patient sample; and green squares indicate the presence of *U. parvum*. Black boxes indicate whether a neonate developed BPD. One sample ran out (gray squares) and could not be tested.

Next, we aimed to determine whether the presence of *U. parvum* creates a distinct inflammatory environment compared to *U. urealyticum*. Here to comparative analysis of the levels of inflammatory cytokines and chemokines in the airway supernatants was performed. Among the five *Ureaplasma*-positive samples, the highest levels of IL-6 and IL-8 were detected in the samples from neonate #2 (*U. urealyticum*) and neonate #3 (*U. parvum*) (Fig. 1). The neutrophil chemoattractant CXCL1 and monocyte attractant CCL2 were highest in the *U. urealyticum*-positive samples neonate #2 and neonate #7, while the neutrophil-activating CXCL5 was highest in *U. parvum*-positive sample neonate #3. This small data set suggests that *U. parvum* and *U. urealyticum* may exist in different inflammatory microenvironments.

### *U. parvum* induces stronger alveolar epithelial immune cell responses than *U. urealyticum*

We observed differences in the inflammatory responses between *U. urealyticum* and *U. parvum* in clinical samples, potentially influenced by different cell types. Since respiratory epithelial cells are the first to encounter *Ureaplasma* spp., we compared the inflammatory responses of these cells to infection by *U. urealyticum* and *U. parvum* in a controlled in vitro model. Supernatants were collected from epithelial cells infected with three different isolates of each species.

**Fig. 2:**
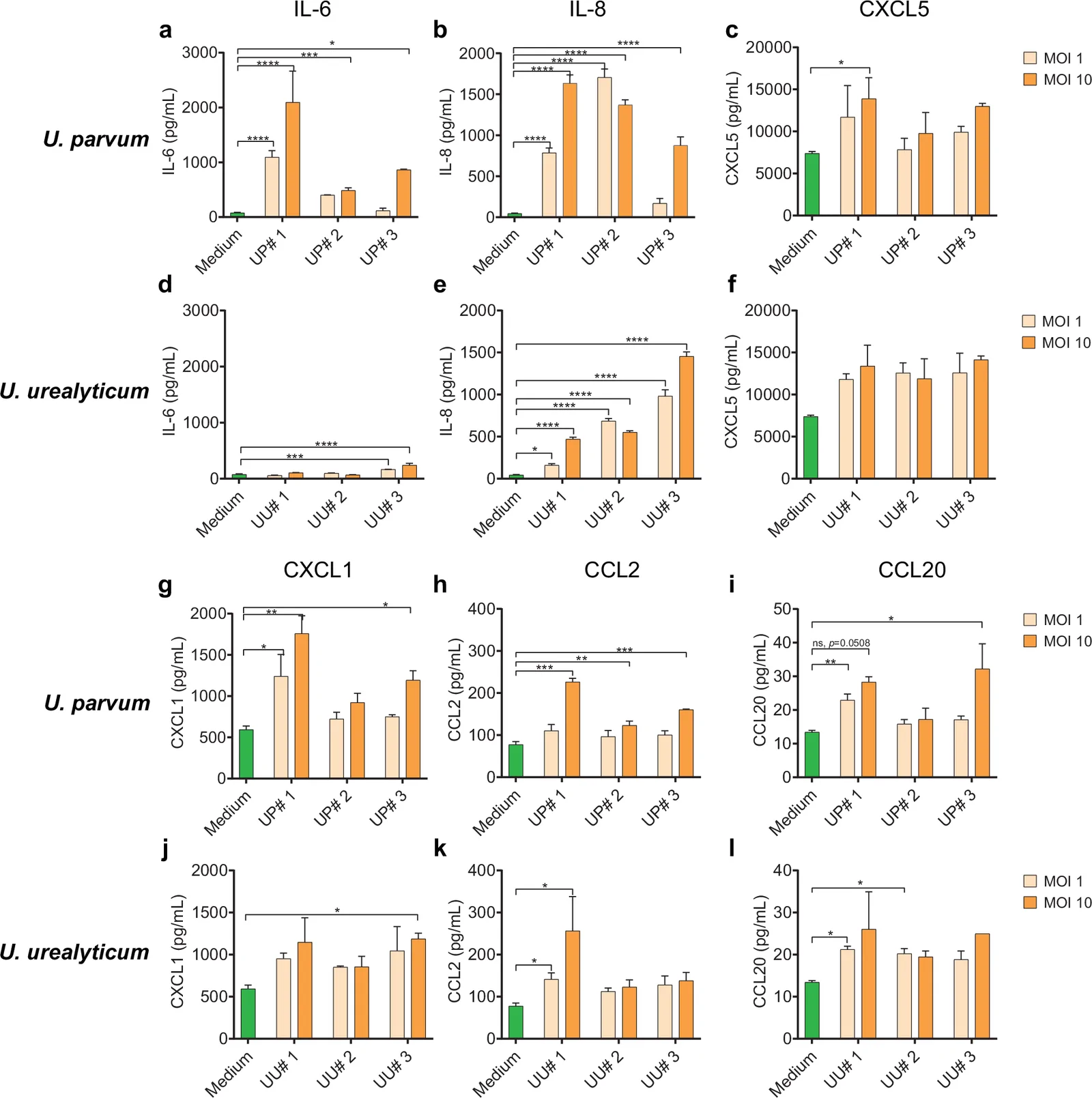
*U. parvum* elicits stronger alveolar epithelial inflammation than *U. urealyticum.* Alveolar epithelial cells were inoculated with three different strains of *U. urealyticum* (UU) or *U. parvum* (UP) at MOI 1 and MOI 10. Three strains of each species were tested: UP#1 = *U. parvum* clinical isolate 2990, UP#2 = *U. parvum* ATCC 700970, UP#3 = *U. parvum* ATCC 27815; UU#1 = *U. urealyticum* ATCC 27618, UU#2 = *U. urealyticum* ATCC 27817, UU#3 = *U. urealyticum* clinical isolate 2983. After 24 h, the levels of pro-inflammatory cytokines and chemokines in these supernatants were determined by **a**, **d** ELISA (IL-6) and **b**, **c**, **e**–**l** LEGEND-plex, respectively. Bars represent the mean ± SD of triplicate cultures. Representative results of three independent experiments. * *p* < 0.05, ** *p* < 0.01, *** *p* < 0.001, and **** *p* < 0.0001 (One-way ANOVA followed by Dunnett’s multiple comparison test).

The production of IL-6 was 5- to 8-fold higher in the supernatants of epithelial cells infected with *U. parvum* strains compared with *U. urealyticum* strains at the same MOI (Figs. 2a, d and S2). Similar observations were made for IL-8, where cytokine production was higher following incubation with *U. parvum* compared to *U. urealyticum*, except in the case for *U. parvum* strain #3 at MOI 1 (Fig. 2b, e). Given that *Ureaplasma* infection of the respiratory tract strongly promotes neutrophil influx,^39,40^ we also determined the levels of neutrophil-attracting and -activating chemokines CXCL5 and CXCL1, in the supernatants. Both *U. parvum* and *U. urealyticum* strains, at MOI 1 and MOI 10, significantly increased the production of CXCL5 (Fig. 2c, f) and CXCL1 (Fig. 2g, j) by epithelial cells compared to uninfected control cells. However, the levels of these chemokines were not significantly different between *U. urealyticum*-inoculated and *U. parvum*-inoculated cultures. All *U. parvum* strains at MOI 10 significantly increased CCL2 production compared to uninfected controls, but this was only observed for strain #1 of the *U. urealyticum* strains (Fig. 2h, k). Infection with either *U. parvum* or *U. urealyticum* strains resulted in low levels of CCL20 (Fig. 2i, l). No differences were found between reference and clinical strains in their ability to induce pro-inflammatory cytokines in alveolar epithelial cells (Fig. 2). These data indicate that *U. parvum* and *U. urealyticum* upregulate expression of different sets of inflammatory mediators in human alveolar epithelial cells.

### *U. urealyticum* infection drives more alveolar cell death than *U. parvum*

**Fig. 3:**
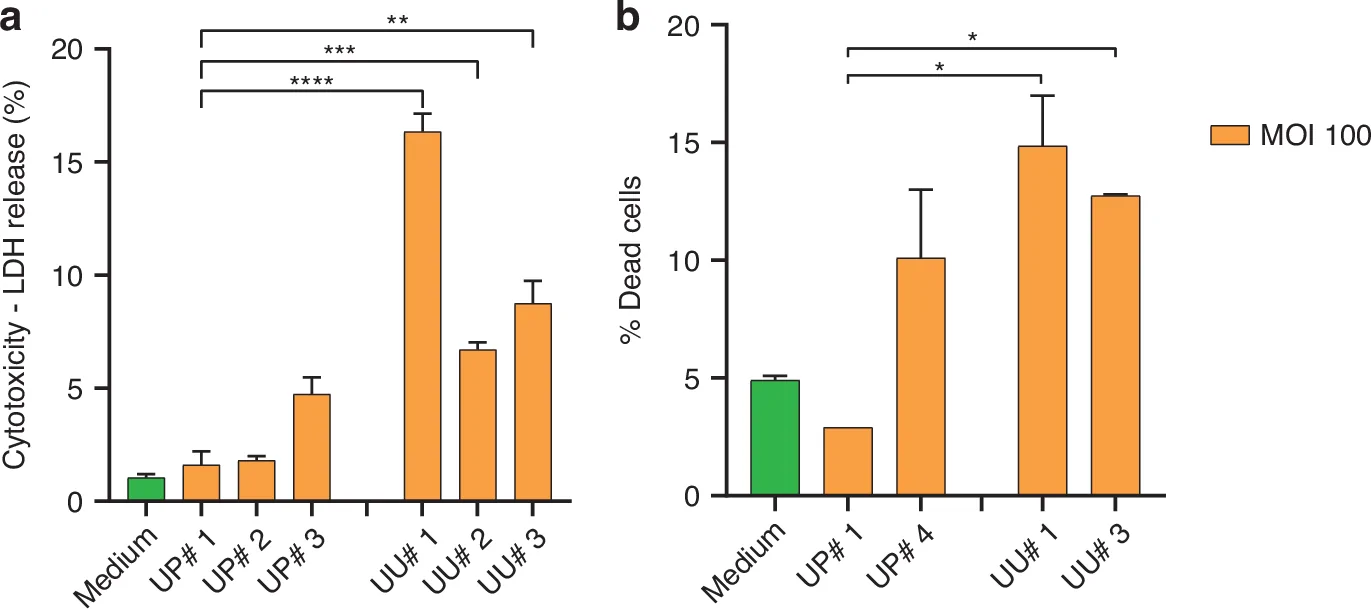
*U. urealyticum* drives more cell death of alveolar epithelial cells than *U. parvum.* Alveolar epithelial cells were inoculated with the indicated *U. urealyticum* (UU) or *U. parvum* (UP) strains at a MOI 100. After a 48 h incubation, cells and supernatants were harvested. **a** Supernatants were analyzed for the amounts of LDH release, which indicates cell death. Bars represent the mean ± SEM of triplicate cultures. **b** Cells were incubated with a LIVE/DEAD marker, and the frequency of dead cells was assessed using flow cytometry. Bars show the mean ± SEM of duplicate cultures. Representative results of three independent experiments. UP#1 = *U. parvum* clinical isolate 2990, UP#2 = *U. parvum* ATCC 700970, UP#3 = *U. parvum* ATCC 27815, UP#4 = *U. parvum* clinical isolate 3763; UU#1 = *U. urealyticum* ATCC 27618, UU#2 = *U. urealyticum* ATCC 27817, UU#3 = *U. urealyticum* clinical isolate 2983. * *p* < 0.05, ** *p* < 0.01, *** *p* < 0.001 and **** *p* < 0.0001 (One-way ANOVA followed by Dunnett’s multiple comparison test).

We subsequently investigated whether the alveolar cell death observed in vivo in *Ureaplasma-*positive airways^24,33^ results from direct *Ureaplasma* infection. First, epithelial cell damage was assessed using an LDH assay. As shown in Fig. 3a, U. *urealyticum* induced more cell damage, leading to greater LDH release than *U. parvum*. The mean percentage of cytotoxicity in *U. urealyticum-*infected cultures was 10.6% ± 1.52, while in *U. parvum-*containing cultures it was 2.7% ± 0.58. LDH release induced by *U. parvum* was not significantly different from that of uninfected control cells. Similar results were obtained when using a viability dye and flow cytometry to assess cell death (Fig. 3b). Infection with *U. urealyticum* at MOI 100 resulted in an average of 13.8% (±1.07) dead cells, while infection with *U. parvum* resulted in 50% fewer dead cells, with an average of 6.5% (±2.39) dead cells.

### Differential effects of *Ureaplasma* species on inflammation and cytotoxicity are maintained and enhanced by *E. coli*-derived LPS

**Fig. 4:**
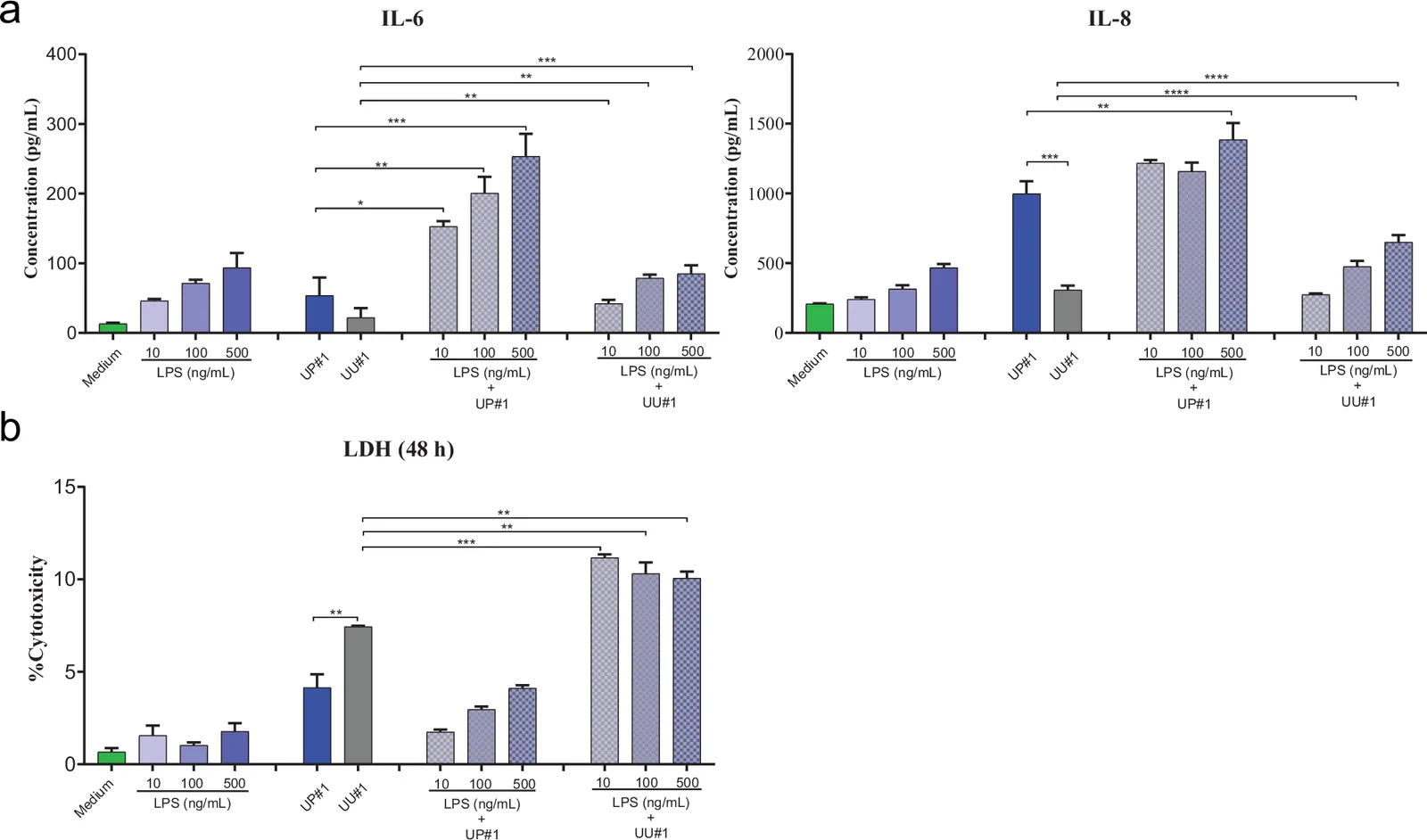
*E. coli*-derived LPS potentiates the differential *Ureaplasma*-induced inflammation and cell death. Alveolar epithelial cells were activated with UP#1 = *U. parvum* clinical isolate 2990 or UU#1 = *U. urealyticum* ATCC 27618 at a MOI 10 or MOI 100 in the absence or presence of indicated concentrations of LPS. **a** After 24 h culture at MOI 10, supernatants were harvested, and the levels of IL-6 and IL-8 were determined by ELISA. **b** After 48 h at MOI 100, cell damage was determined by quantifying the amounts of LDH released into the supernatants. Bars show the mean ± SEM of triplicate cultures. Representative results of two independent experiments. * *p* < 0.05, ** *p* < 0.01, *** *p* < 0.001, and **** *p* < 0.0001 (One-way ANOVA followed by Dunnett’s multiple comparison test).

Since *E. coli* was co-detected in three of the five *Ureaplasma*-positive neonatal samples, we speculated that the presence of *Ureaplasma* might prime the innate immune system to mount a stronger response to other bacterial infections. To test this, we performed infection assays in the presence of LPS derived from *E. coli*. The concentration of LPS used in these assays reflected the mean number of *E. coli* bacteria detected in the neonatal samples. The administration of LPS to epithelial cells resulted in a concentration-dependent increase in IL-6 and IL-8 production (Fig. 4a). The presence of a low amount of LPS, i.e., 10 ng/mL, increased IL-6 levels induced by *U. parvum* by three-fold, while IL-8 increased only slightly (Fig. 4a). IL-8 was significantly increased when higher amounts of LPS, i.e., 500 ng/mL, were present. Co-activation of alveolar epithelial cells with *U. urealyticum* and LPS also increased IL-6 and IL-8 secretion, but the cytokine levels did not reach those induced by *U. parvum*. In fact, they remained 2.5 to 4.5-fold lower compared to the levels measured in *U. parvum*-infected cultures (Fig. 4a; using 10 ng/mL LPS, mean IL-6: 42.3 vs 152.6 pg/mL; and mean IL-8: 273.9 vs 1215.6 pg/mL. Using 100 ng/mL LPS, mean IL-6: 78.6 vs 200.5 pg/mL; and mean IL-8: 475.7 vs 1157.9 pg/mL). *U. urealyticum*-induced cell cytotoxicity was enhanced by LPS: a mean of 11.2% cytotoxicity was measured in the presence of LPS versus 7.4% in its absence (Fig. 4b). In contrast, addition of LPS to *U. parvum*-infected cultures did not increase cell cytotoxicity, with 1.7% compared to 4.1% without LPS (Fig. 4b). These data indicate that the presence of other microorganisms, such as *E. coli*, potentiates *U. parvum*-induced inflammation and *U. urealyticum*-induced cell death.

### *Ureaplasma* infection induced oxidative stress

**Fig. 5:**
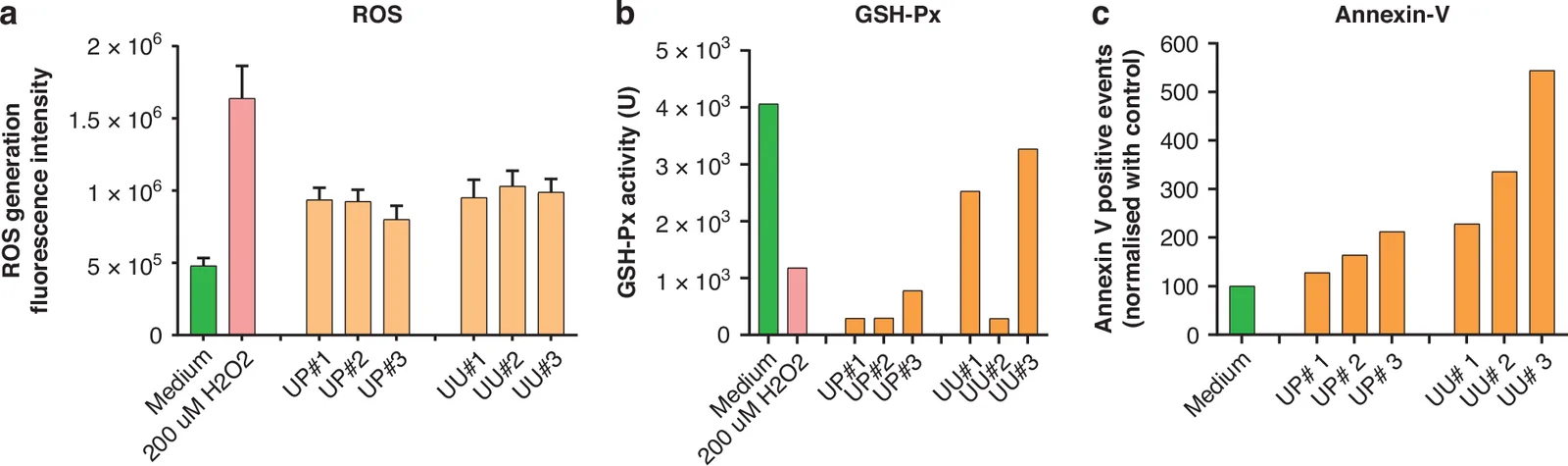
*Ureaplasma* infection results in oxidative stress. Alveolar epithelial cells were activated with indicated *U. urealyticum* (UU) or *U. parvum* (UP) isolates at a MOI 10 or 100. UP#1 = *U. parvum* clinical isolate 2990, UP#2 = *U. parvum* ATCC 700970, UP#3 = *U. parvum* ATCC 27815; UU#1 = *U. urealyticum* ATCC 27618, UU#2 = *U. urealyticum* ATCC 27817, UU#3 = *U. urealyticum* clinical isolate 2983. **a** To assess ROS generation, cells were stained with DCFDA, and after 3 h culture with *Ureaplasma* bacteria at MOI 10, ROS was detected by fluorescence spectroscopy with excitation/emission at 485/535 nm. **b** Cells were cultured with *Ureaplasma* bacteria at MOI 100, and after 24 h the activity of the antioxidant GSH-Px was determined using a commercial kit, and **c** the induction of apoptosis was determined by staining cells with Annexin-V antibody. Cells were analyzed by fluorescence microscopy and Annexin-V positive events were quantified using ImageJ. Representative results of two independent experiments. (One-way ANOVA followed by Dunnett’s multiple comparison test).

Inflammation and cell death are closely linked to oxidative stress. Since oxidative stress is also frequently observed in BPD,^41^ we next examined the potential of *U. parvum* and *U. urealyticum* to induce oxidative stress by measuring the generation of ROS, the activity of MDA, and the concentration of the antioxidant GSH-Px. Infection of epithelial cells with either *U. parvum* or *U. urealyticum* resulted in robust generation of ROS (Fig. 5a). Vice versa, the levels of GSH-Px were reduced upon infection with *U. parvum* and *U. urealyticum*, with most strong effects observed in *U. parvum* infected cultures (Fig. 5b). GSH-Px activity is higher in *U. urealyticum* infected lung cells than in *U.parvum* infected cells. Interestingly, incubation with either *Ureaplasma* species did not result in lipid peroxidation, as no MDA could be detected (data not shown). This finding indicates that the cell death induced by *U. urealyticum* is not ferroptosis. Instead, evaluation of Annexin-V revealed that infection with *U. urealyticum* resulted in the induction of apoptosis. A two- to five-fold increase in Annexin-V positive events was observed in *U. urealyticum* containing cultures compared to control and *U. parvum* cultures (Fig. 5c). The limited occurrence of apoptotic events in *U. parvum* cultures is consistent with the observations made on LDH and cell death (Fig. 3a). Together, these data suggest that oxidative stress triggers the inflammation and cell death induced by *U. parvum* and *U. urealyticum*, respectively.

### Oxidative stress, inflammation, and cell death in alveolar epithelial cells is triggered by a secreted factor from *Ureaplasma*

**Fig. 6:**
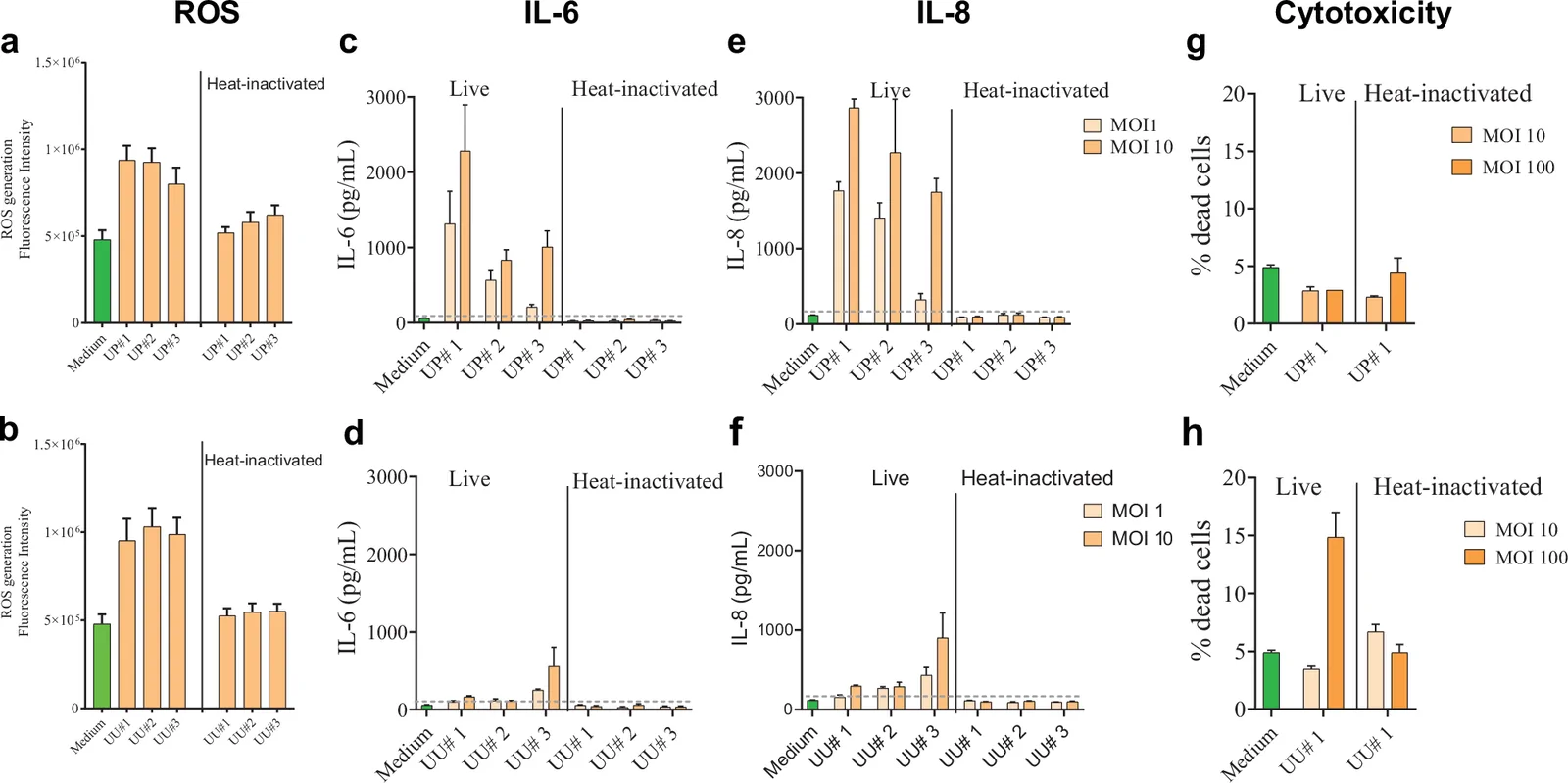
Alveolar oxidative stress, inflammation and cell death are triggered by soluble factors secreted by *Ureaplasma.* Alveolar epithelial cells were inoculated with heat-inactivated or live *U. urealyticum* or *U. parvum* strains at indicated MOI. Cells were stained with DCFDA to determine ROS generation after 3 h (**a**, **b**). Culture supernatants were harvested after 24 h and 48 h incubation. (**c**, **d**) IL-6 and (**e**, **f**) IL-8 levels in the culture supernatants were determined by ELISA. **g**, **h** Cytotoxicity was determined by quantifying the amount of LDH. Bars show the mean ± SD of triplicate cultures, and one representative of two independent experiments is shown. UP#1 = *U. parvum* clinical isolate 2990, UP#2 = *U. parvum* ATCC 700970, UP#3 = *U. parvum* ATCC 27815; UU#1 = *U. urealyticum* ATCC 27618, UU#2 = *U. urealyticum* ATCC 27817, UU#3 = *U. urealyticum* clinical isolate 2983. * *p* < 0.05, ** *p* < 0.01, *** *p* < 0.001, and **** *p* < 0.0001 (One-way ANOVA followed by Dunnett’s multiple comparison test).

The inflammatory and cell-damaging effects observed in airway epithelial cells following *Ureaplasma* infection could be due to direct membrane-membrane interactions or the secretion of factors by *Ureaplasma* during proliferation in the epithelial cells.^42,43^ To test the latter, we heat-inactivated the *Ureaplasma strains*, making them incapable to secrete products. Infection of alveolar cells with either heat-inactivated *U. parvum* or heat-inactivated *U. urealyticum* did not lead to the generation of significant amounts of ROS (Fig. 6a, b). Similarly, infection of epithelial cells with heat-inactivated bacteria did not induce significant production of IL-6 (Fig. 6c, d) or IL-8 (Fig. 6e, f). The levels of ROS in the epithelial cells and the cytokines in the culture supernatants were comparable to those in the medium control cultures. Both inflammatory cytokines were only produced by alveolar epithelial cells in response to live, proliferating *Ureaplasma* spp., with the highest levels of IL-6 and IL-8 found in *U. parvum*-infected cultures (Fig. 6c, e). Analysis of supernatants for LDH release indicated that *U. urealyticum*-induced cytotoxicity was also dependent on factors released by live, active bacteria (Fig. 6h). While infection with live *U. urealyticum* at MOI 100 resulted in 15% dead cells, the frequency of dead cells in cultures with heat-inactivated bacteria was significantly reduced to the level of medium control wells (Fig. 6h). *U. parvum* strains did not induce cell death (Fig. 6g). Together, these findings suggest that the induction of inflammation and cell damage in epithelial cells is dependent on the proliferation and factors secreted by live *U. parvum* and *U. urealyticum*.

## Discussion

In this study, we provide novel insights into the differences in immunomodulatory capacities between the two *Ureaplasma* species associated with the development of BPD in premature infants. A direct comparison of *U. urealyticum* and *U. parvum* in a controlled in vitro infection model demonstrated that both *Ureaplasma* species may contribute to inflammation and cell damage as can be observed in BPD, yet through different mechanisms. We observed a stronger pro-inflammatory mediator response in alveolar epithelial cells infected with *U. parvum* compared to *U. urealyticum*. Additionally, more epithelial cell death occurred after incubation with *U. urealyticum* than with *U. parvum*. These damaging effects appear to be triggered by oxidative stress and are largely dependent on soluble factors secreted by *Ureaplasma*. This is evidenced by the observation that heat-inactivated *Ureaplasma* isolates induced minimal alveolar epithelial cell responses compared to live isolates.

In our cohort of premature neonates with respiratory insufficiency and need for mechanical ventilation, 5 of 25 (20%) neonates had *Ureaplasma*-positive tracheal aspirates, and all but one eventually developed BPD. This frequency aligns with observational studies showing that *Ureaplasma* presence in neonatal airways is associated with BPD.^20,44–46^ Early life *Ureaplasma* spp. exposure and prolonged mechanical ventilation may contribute to BPD development,^45^ with factors such as serovar virulence, colonization duration*, Ureaplasma* load, and co-colonization with other bacteria, potentially influencing outcomes.^44^

Besides *Ureaplasma* spp., also *K. pneumoniae*, *S. aureus*, and *E. coli* were detected in the lungs of infants, but only *E. coli* was found in *Ureaplasma*-positive infants. GBS was not detected in any neonatal airway sample, possibly due to the small cohort. Alternatively, some newborns may have detectable gastro-intestinal carriage without oropharyngeal GBS presence.^47^ We cannot fully attribute this to a negative GBS carriage status in the mothers. Retrospective analysis indicated that maternal GBS carriage status was negative in 52%, and unknown in 48%, as routine maternal GBS screening is not conducted in the Netherlands.

Analysis of inflammatory mediators in the tracheal aspirates revealed distinct profiles in *U. urealyticum-*positive airways compared to *U. parvum*-positive airways, with the most notable differences observed in CXCL1, CXCL5, CCL20, and CCL2. The chemokines CXCL1, CCL20, and CCL2 were most elevated in *U. urealyticum-*positive samples, while CXCL5 was dominant in *U. parvum*-positive samples. These differences may reflect the recruitment and activation of different cell types at the sites of *U. urealyticum* versus *U. parvum* infection, and consequently, a distinct type of inflammation. At *U. urealyticum* infection sites, IL-8 and CCL2, derived from alveolar epithelial cells, mediate the recruitment of neutrophils and monocytes. These cells further amplify the inflammation process by secreting CXCL1 and CCL20, which recruit additional leukocytes.^48^ While CCL20 primarily recruits CCR6^+^ effector B and T cells, CXCL1 facilitates another wave of neutrophil recruitment and activation for microbial killing. These findings are consistent with previous studies in humans and experimental animal models, which showed that *Ureaplasma*-positive preterm newborns had higher concentrations of pro-inflammatory mediators, such as IL-8, IL-1β, IL-6, and CXCL1, as well as elevated numbers of neutrophils in their airway samples compared to *Ureaplasma*-negative newborns.^11,28,39,40^

In the *U. parvum*-positive airways, other than high IL-6 and IL-8, high levels of CXCL5 were detected, particularly in patient 3. During bacterial challenge in the airways, CXCL5 is secreted by activated alveolar type II epithelial cells to mediate optimal recruitment and activation of neutrophils at the infection site. However, it has been shown that CXCL5 can impede this process during severe airway infections with Gram-negative bacteria such as *E.coli* and *K. pneumoniae*.^49,50^ CXCL5 can regulate the sequestration in blood and here with bioavailability of CXCL1 and CXCL2, two other neutrophil attractants. In case of high CXCL5 levels, sequestration of CXCL1 and CXCL2 is prevented, resulting in high levels of these chemokines in blood and hereby inversing the chemokine gradient between blood and infected tissue. Consequently, recruitment and activation of neutrophils at the infection site are impaired. Interestingly, *E. coli* was co-detected with *U. parvum* in the tracheal aspirate of patient 3. Whether neutrophil influx into the airways of this patient was hampered via the above-described CXCL5 mechanism is unknown, as analysis of the immune composition of the tracheal aspirates was beyond the scope of our explorative study.

Using an in vitro infection model with human alveolar epithelial cells we determined whether differences between *U. urealyticum* and *U. parvum* pathogenicity already arise at the epithelial level, the first barrier for pulmonary pathogens. Infection with *U. parvum* resulted in five-to-eight-fold higher IL-6 and IL-8 production by human epithelial cells than with *U. urealyticum*. In contrast, no significant differences in CXCL1, CXCL5, and CCL2 levels as found in our neonatal airway samples were detected in vitro. In case of CXCL1, this is likely explained by the fact that this chemokine is mainly expressed by neutrophils and macrophages in response to *Ureaplasma*. These cells were not present in our in vitro infection model. Also, in vivo, other bacteria such as *E. coli* can be present. The in vitro experiments testing the influence of *E. coli* on *Ureaplasma* infection by adding *E. coli*-derived LPS indicated that the specific responses induced by either *U. parvum* (i.e., high levels of pro-inflammatory mediators) or *U. urealyticum* (i.e., cytotoxicity) were further enhanced. Comparable enhancement of LPS-induced pro-inflammatory cytokine production by *Ureaplasma* was shown in studies using neonatal monocytes.^34,51^ Here, co-stimulation of neonatal monocytes with *Ureaplasma* and LPS maintained and aggravated pro-inflammatory responses. In one of these in vitro studies, responses between a *U. parvum* serotype 3 strain and a *U. urealyticum* serotype 8 strain were compared. However, in contrast to our findings, no major overall differences in the cytokine responses induced by these two strains were observed. This could be related to the fact that a different host cell type (i.e., monocytes versus alveolar epithelium) was used than in our study. Alternatively, it may be due to the fact that only one strain of each species was tested. In our study, we tested three different isolates of each species and observed that for certain inflammatory mediators, one of the *U. urealyticum* isolates (e.g., *U. urealyticum* #3-2983) induced higher levels than the other isolates tested. Nevertheless, our data obtained ex vivo in neonatal tracheal aspirates and in vitro infection model are in line with previous speculations that *Ureaplasma* infection might affect immunity to a second microorganism by exacerbating inflammation and promote tissue injury by inducing cell cytotoxicity. Of note, *Ureaplasma*-positive neonate #2 was the one patient in whom *E. coli* was not co-detected. Despite that, high levels of pro-inflammatory mediators were measured in the tracheal aspirate. Of the five *Ureaplasma*-positive neonates, neonate #2 had been longest on a ventilator (i.e., 84 days versus 5-19 days, respectively). There is accumulating evidence that the sensitivity of the neonatal lung to stressors such as mechanical ventilation is increased by pulmonary infection and inflammation.^52,53^ Indeed, in preterm infants, prolonged ventilation additive to colonization with *Ureaplasma* spp. has been shown to confer an increased risk of severe chronic lung injury and/or development of BPD.^23,32,54,55^

Epithelial cultures infected with *U. urealyticum* showed more cell death than those infected with *U. parvum. Ureaplasma* presence may damage host cells through inflammation, either via membrane-associated molecules or via soluble factors.^4,56,57^ A key membrane-associated protein in *Ureaplasma* spp., multiple-banded antigen (MBA), triggers production of inflammatory mediators by binding to TLR2/1 and TLR2/6 on alveolar epithelial cells and macrophages, leading to IL-1β and TNF production, which are linked with cell damage and lung fibrosis.^58^ However, the observation that heat-inactivation of the *Ureaplasma* strains abolished their ability to induce inflammation in alveolar epithelial cells suggests that soluble factors, rather than MBA, are the primary contributors to pathogenicity. The factors in question comprise ammonia, hydrogen peroxidase, and phospholipases. Ammonia is produced from urease metabolism by *Ureaplasma* spp., which can cause toxicity to host tissues due to a shift in pH. Altered pH and increased ammonia concentrations of the amniotic fluid have been linked to intra-uterine *Ureaplasma* infections,^59^ and the ammonia liberated by *Ureaplasma* may contribute to chronic tissue damage in the fetal lung.^60^ Moreover, it has been proposed that ammonia can react with water to form ammonium hydroxide, which could in turn promote injury and inflammation of the mucosa. Membrane peroxidation and phospholipid degradation by hydrogen peroxidase and phospholipases also play a role in cell damage. The results obtained on oxidative stress markers suggest that the inflammatory and cytotoxic effects on human lung cells following infection with, respectively, *U. parvum* and *U. urealyticum* might be mediated by ROS. However, the involvement of this pathway might be to a different extent in *U. parvum* than *U. urealyticum* infections, as shown by the higher activity of the antioxidant GSH-Px in *U.* *urealyticum-*infected cells compared to *U. parvum* infected cells. The higher GSH-Px activity was observed despite a seemingly equal induction of ROS. Further investigation is required to ascertain the precise molecular pathways that lead to oxidative stress-induced inflammation and apoptosis by various *U. parvum* and *U. urealyticum* isolates.

Our analysis of the clinical samples has some limitations. First, we lack information on the duration of *Ureaplasma* exposure in the respiratory tract of preterm neonates, both intra-uterine and after birth. Longer exposure is likely to induce immune cell influx, leading to a cascade of responses to *Ureaplasma*. This exposure time likely varies among neonates, challenging the comparison of analyses. The decision to collect tracheal aspirate samples was made by the treating physician, and the obtained tracheal aspirate volume was not standardized, varying depending on the treating physician. Lastly, the number of subjects included in this explorative study was small.

In summary, *U. parvum* and *U. urealyticum* species exhibit distinct effects on the immune responses and apoptosis of alveolar cells in vitro. While alveolar epithelial cells infected with *U. parvum* resulted in a stronger pro-inflammatory mediator response compared to *U. urealyticum*, more epithelial cell death occurred after incubation with *U. urealyticum* than with *U. parvum*. These *Ureaplasma*-induced inflammatory responses appear to be mediated via ROS and driven by secreted factors as responses induced by heat-inactivated bacteria were significantly lower than those by live bacteria. The development of BPD in infants associated with the presence of *Ureaplasma* spp. may result from various etiologies, as different cytokine and chemokine profiles were also observed in clinical tracheal samples. These findings contribute to the understanding of the inflammatory processes and cell death at the site of *Ureaplasma* infections, providing insights for developing targeted therapies or preventive strategies for BPD development.

## Supplementary information


Supplementary information


## Data Availability

The data that support the findings of this study are available from the corresponding author upon reasonable request.
