# Publisher Correction: *Ureaplasma parvum* and *Ureaplasma urealyticum* induce distinct types of inflammation in neonates and human epithelial cell models

**DOI:** 10.1038/s41390-025-04547-3

**Published:** 2025-10-28

**Authors:** Hongzhen Zhu, Pol Oliveras-Julià, Gea F. Hasperhoven, Luca L. van Leeuwen, Ad C. J. M. de Bruijn, Marijn C. Verwijs, Annemarie M. C. van Rossum, René F. Kornelisse, Kim Stol, Wendy W. J. Unger

**Affiliations:** 1 Department of Pediatrics, Laboratory of Pediatrics, Erasmus MC University Medical Center - Sophia Children’s Hospital, Rotterdam, The Netherlands; 2 Department of Neonatal and Pediatric Intensive Care, Division of Neonatology, Erasmus MC University Medical Center - Sophia Children’s Hospital, Rotterdam, The Netherlands; 3 Department of Pediatrics, Division of Pediatric Infectious Diseases and Immunology, Erasmus MC University Medical Center - Sophia Children’s Hospital, Rotterdam, The Netherlands; 4 Department of Pediatrics, Division of Pediatric Infectious Diseases and Immunology, Radboud University Medical Center, Amalia Children’s Hospital, Nijmegen, The Netherlands

Correction to: *Pediatric Research* 10.1038/s41390-025-04415-0; published online 03 October 2025

Due to a typesetting mistake, in Figures 2, 4 and 6 the abbreviation to denote UU has been replaced by UP, which was incorrect. The publishers would like to apologize for this mistake. The figure have been corrected.

Former figures 2:
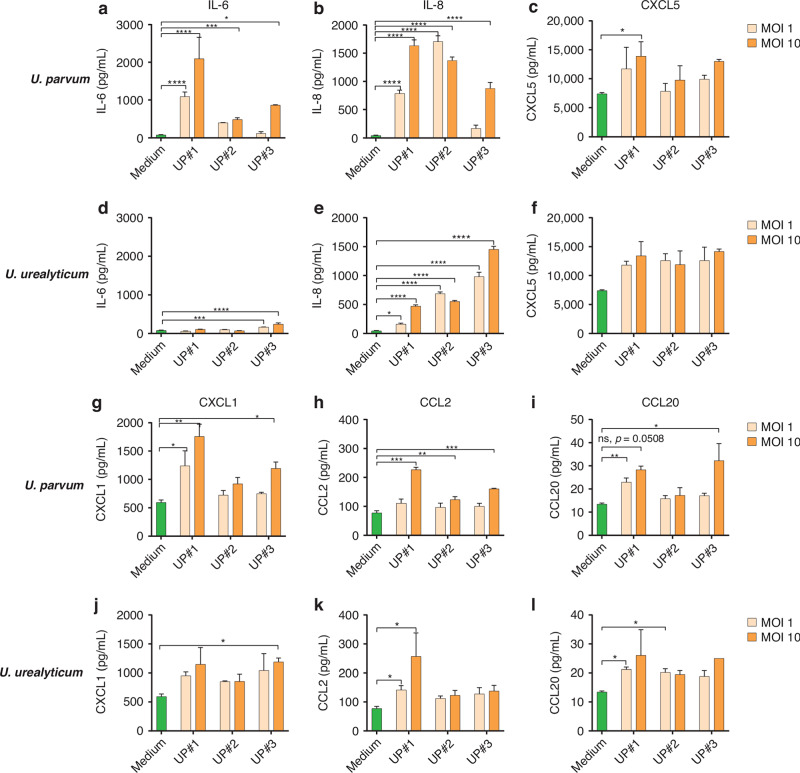
Corrected Figure 2:
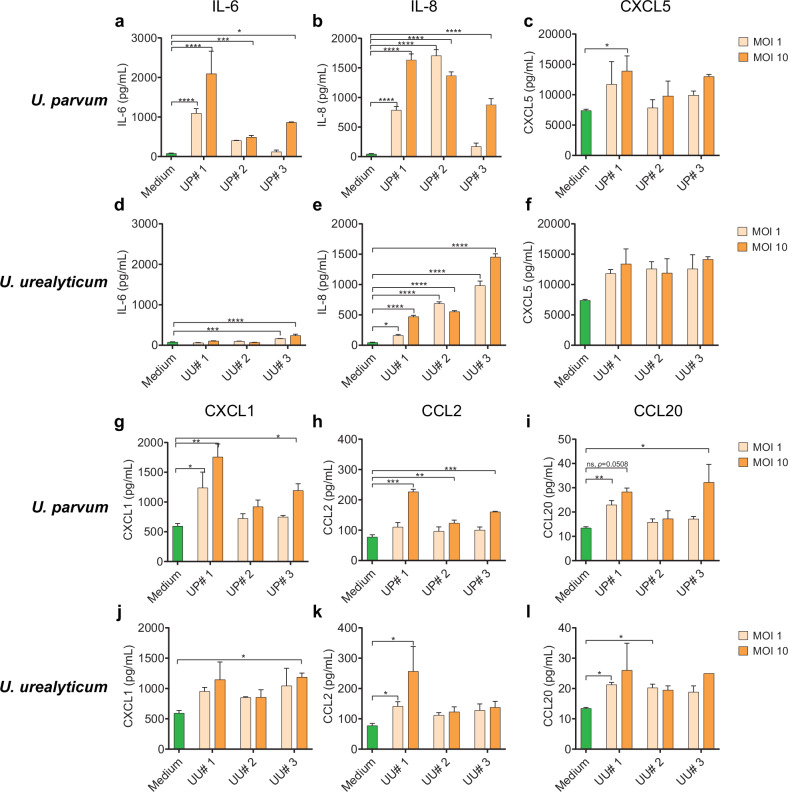
Former Figure 4:
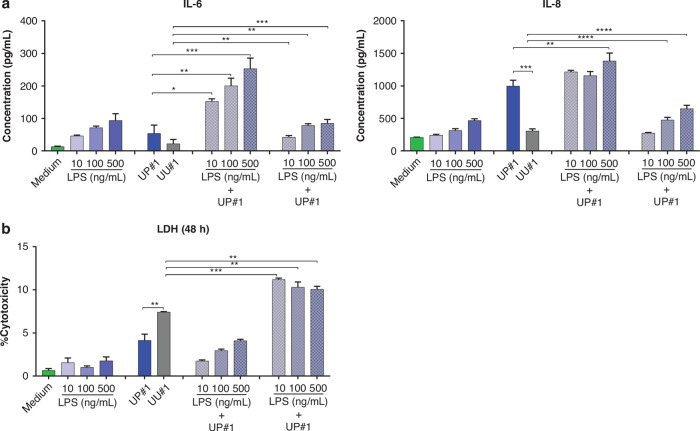


Corrected figures 4:
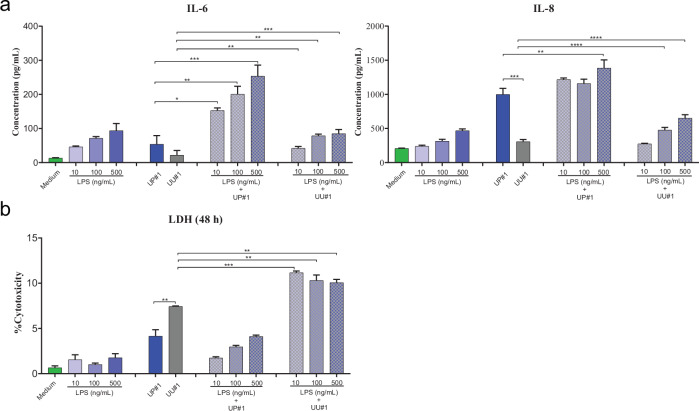
Former Figure 6:
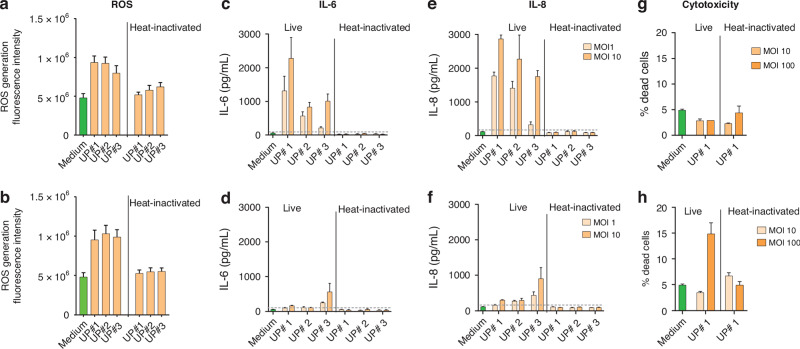
Corrected Figure 6:
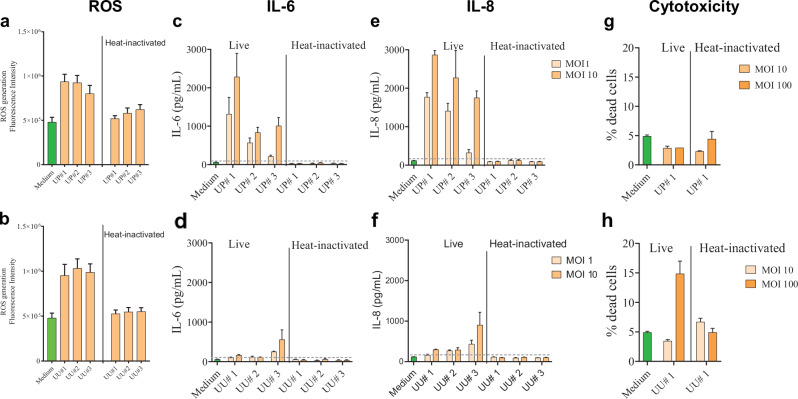


The original article has been corrected.

