# Supplementary material for: Ureaplasma parvum and Ureaplasma urealyticum induce distinct types of inflammation in neonates and human epithelial cell models
Source: Pediatr Res. 2025 Oct 3;100(1):410–9. doi: 10.1038/s41390-025-04415-0 (PMC13524813; doi:10.1038/s41390-025-04415-0)
Supplement: Supplementary file 1 [file 41390_2025_4415_MOESM1_ESM.pdf]

Supplemental Material

Supplemental Figure S1 Visualization of bacteria in neonatal tracheal aspirates

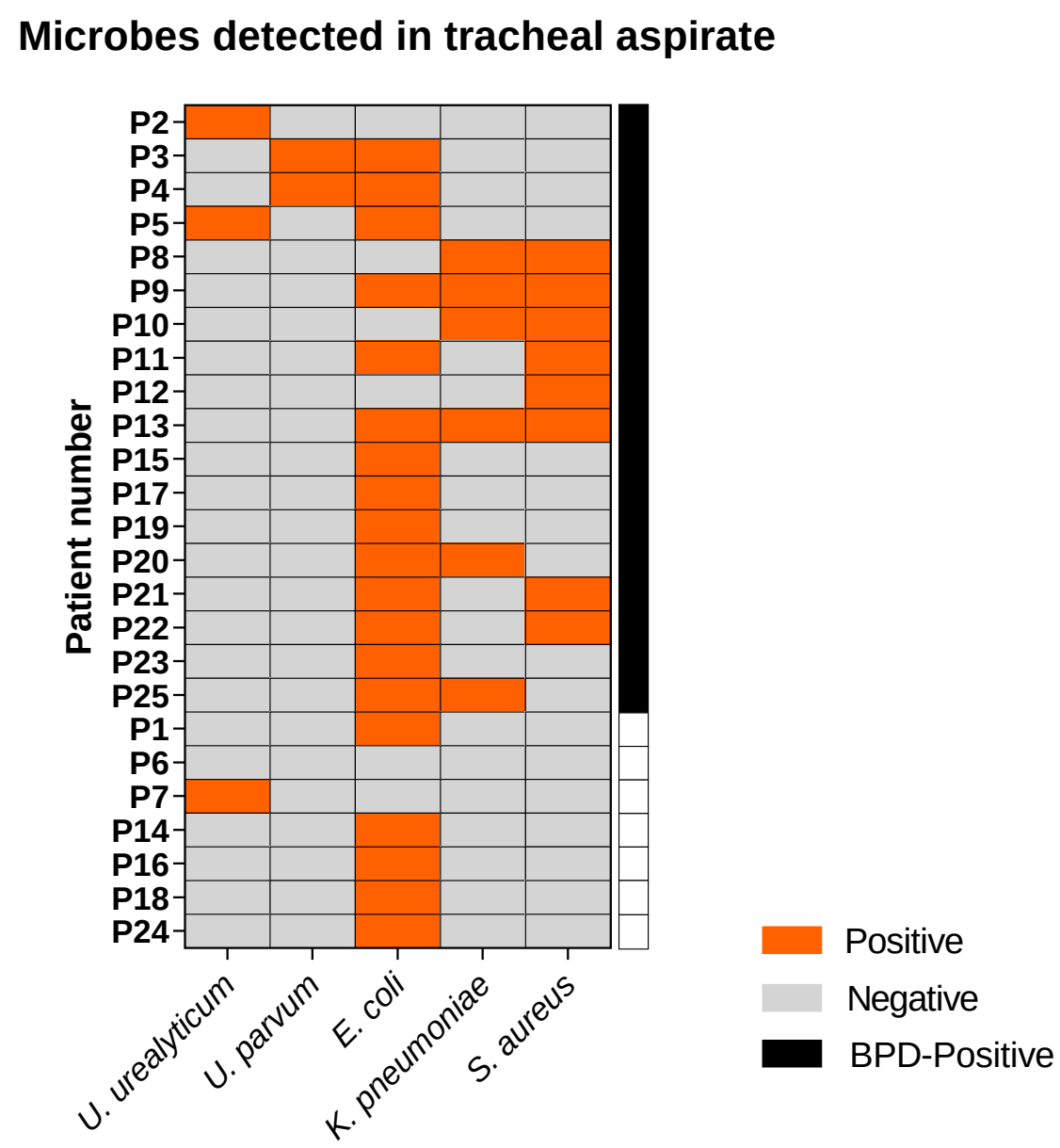

Supplemental Fig S1 Tracheal aspirates from 25 preterm neonates were analyzed for presence of *U. urealyticum*, *U. parvum, E. coli*, *K. pneumoniae*, *S. aureus* and GBS using PCR. The positive and negative results for each bacterium are indicated for each patient. One sample was ran out (P6) and could not be tested.

Supplemental Figure S2 The amounts of IL-6 induced by *U. urealyticum*

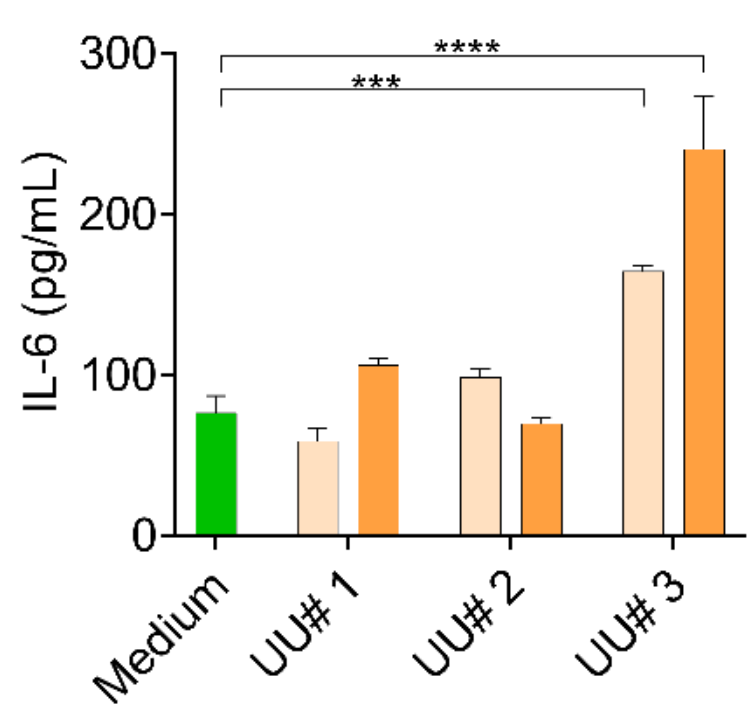

Supplemental Fig S2 The amounts of IL-6 induced by *U. urealyticum* determined by ELISA. Bars represent the mean ± SD of triplicate cultures. Representative results of three independent experiments. * p<0.05, ** p<0.01, *** p<0.001 and **** p<0.0001 (One-way ANOVA followed by Dunnett's multiple comparison test).

Supplemental Table S1 The primers used for the neonatal tracheal aspirate polymerase chain reaction assays.

**Multiplex PCR**				
**Pathogen**	**Primer/probe**	**Sequence**	**Target gene**	**Reference**
Ureaplasma urealyticum	Forward primer<br>Reverse primer<br>Probe:	5'-AAGGTCAAGGTATGGAAGATCCAA-3'<br>5'-TTCCTGTTGCCCCTCAGTCT-3'<br>5'-ACCACAAGCACCTGCTACGATTTGTTC-3'	*Urease* gene	1
U. parvum	Forward primer<br>Reverse primer<br>Probe:	5'-AAGGTCAAGGTATGGAAGATCCAA-3'<br>5'-TTCCTGTTGCCCCTCAGTCT-3'<br>5'-TCCACAAGCTCCAGCAGCAATTTG-3'	*Urease* gene	1
Escherichia coli	Forward primer<br>Reverse primer<br>Probe:	5'-CATGCCGCGTGTATGAAGAA-3'<br>5'-CGGGTAACGTCAATGAGCAAA-3'<br>5'-TTCAGCGGGGAGGAAGGGAGTAAAGTTAATA-3'	16S rDNA	2
Klebsiella pneumoniae	Forward primer<br>Reverse primer<br>Probe:	5'-CGAAACCGCTCGTAAACACA-3'<br>5'-AGGAAGCGTTGGAAACGATG-3'<br>5'-GTTCTGACTGGCCCGTCCAAAGA-3'	*gap* A gene	3
Streptococcus agalactiae	Forward primer<br>Reverse primer<br>Probe:	5'-TTCACCAGCTGTATTAGAAGTACATGC-3'<br>5'-CCCTGAACATTATCTTTGATATTTCTCA-3'<br>5'-CAAGCCCAGCAAATGGCTCAAAAGCT-3'	*cfb* gene	4
**Singleplex PCR**				
**Pathogen**	**Primer/probe**	**Sequence**		
Staphylococcus aureus	Forward primer<br>Reverse primer<br>Probe:	5'-CATCGGAAACATTGTGTTCTGTATG-3'<br>5'-TTTGGCTGGAAAATATAACTCTCGTA-3'<br>5'-AAGCCGTCTTGATAATCTTTAGTAGTACCGAAGCTGGT-3'	Sa442	5
	**Universal primers**	**Sequence**		
**All bacteria**	Forward primer<br>Reverse primer	5'-AGAGTTTGATCCTGGCTCAG-3'<br>5'-CTGCTGCCTCCCGTAG-3'	16S rRNA	6
	**Primer/probe**	**Sequence**		
**Internal Control PhHV-1**	Forward primer<br>Reverse primer<br>Probe	5'- GGGCGAATCACAGATTGAATC-3'<br>5'-GCGGTTCCAAACGTACCAA-3'<br>5'-TTTTTATGTGTCCGCCAC CATCTGGATC-3'	*gB* gene	7

References:

1 Yi, J., Yoon, B. H. & Kim, E. C. Detection and Biovar Discrimination of Ureaplasma Urealyticum by Real-Time Pcr. *Mol Cell Probes* **19**, 255-260 (2005).

2 Huijsdens, X. W. et al. Quantification of Bacteria Adherent to Gastrointestinal Mucosa by Real-Time Pcr. *J Clin Microbiol* **40**, 4423-4427 (2002).

3 Ahn, C., Yoon, S. S., Yong, T. S., Jeong, S. H. & Lee, K. The Resistance Mechanism and Clonal Distribution of Tigecycline-Nonsusceptible Klebsiella Pneumoniae Isolates in Korea. *Yonsei Med J* **57**, 641-646 (2016).

4 van den Brand, M. et al. Development of a Multiplex Real-Time Pcr Assay for the Rapid Diagnosis of Neonatal Late Onset Sepsis. *J Microbiol Methods* **106**, 8-15 (2014).

5 Peters, R. P. et al. Quantitative Detection of Staphylococcus Aureus and Enterococcus Faecalis DNA in Blood to Diagnose Bacteremia in Patients in the Intensive Care Unit. *J Clin Microbiol* **45**, 3641-3646 (2007).

6 Waldeisen, J. R., Wang, T., Mitra, D. & Lee, L. P. A Real-Time Pcr Antibiogram for Drug-Resistant Sepsis. *PLoS One* **6**, e28528 (2011).

7 Nassuuna, J. et al. Helminth Driven Gut Inflammation and Microbial Translocation Associate with Altered Vaccine Responses in Rural Uganda. *NPJ Vaccines* **10**, 56 (2025).
